# BMP-6 promotes type 2 immune response during enhancement of rat mandibular bone defect healing

**DOI:** 10.3389/fimmu.2023.1064238

**Published:** 2023-02-10

**Authors:** Logan F. McColl, Xizhao Chen, Michael D. Solga, Kailo Schlegel, Sean P. Haughey, Peter I. Lobo, Kristen Fread, Eli Zunder, Ryan Cha, Stephen Park, J. Jared Christophel, Quanjun Cui, Abhijit S. Dighe

**Affiliations:** ^1^ Department of Orthopedic Surgery, University of Virginia Health System, Charlottesville, VA, United States; ^2^ Flow Cytometry Core Facility, University of Virginia, Charlottesville, VA, United States; ^3^ Department of Nephrology, University of Virginia Health System, Charlottesville, VA, United States; ^4^ Department of Biomedical Engineering, University of Virginia, Charlottesville, VA, United States; ^5^ Department of Otolaryngology–Head and Neck Surgery, University of Virginia Health System, Charlottesville, VA, United States

**Keywords:** BMP, macrophages, T cells, fracture healing, stem cells, type 2 immune response

## Abstract

**Introduction:**

Bone morphogenetic proteins (BMPs) are used as key therapeutic agents for the treatment of difficult fractures. While their effects on osteoprogenitors are known, little is known about their effects on the immune system.

**Methods:**

We used permutations of BMP-6 (B), vascular endothelial growth factor (V), and Hedgehog signaling pathway activator smoothened agonist (S), to treat a rat mandibular defect and investigated healing outcomes at week 8, in correlation with the cellular landscape of the immune cells in the fracture callus at week 2.

**Results:**

Maximum recruitment of immune cells to the fracture callus is known to occur at week 2. While the control, S, V, and VS groups remained as nonunions at week 8; all BMP-6 containing groups - B, BV, BS and BVS, showed near-complete to complete healing. This healing pattern was strongly associated with significantly higher ratios of CD4 T (CD45^+^CD3^+^CD4^+^) to putative CD8 T cells (CD45^+^CD3^+^CD4^-^), in groups treated with any permutation of BMP-6. Although, the numbers of putative M1 macrophages (CD45^+^CD3^-^CD11b/c^+^CD38^high^) were significantly lower in BMP-6 containing groups in comparison with S and VS groups, percentages of putative - Th1 cells or M1 macrophages (CD45^+^CD4^+^IFN-γ^+^) and putative – NK, NKT or cytotoxic CD8T cells (CD45^+^CD4^-^IFN-γ^+^) were similar in control and all treatment groups. Further interrogation revealed that the BMP-6 treatment promoted type 2 immune response by significantly increasing the numbers of CD45^+^CD3^-^CD11b/c^+^CD38^low^ putative M2 macrophages, putative - Th2 cells or M2 macrophages (CD45^+^CD4^+^IL-4^+^) cells and putative – mast cells, eosinophils or basophils (CD45^+^CD4^-^IL-4^+^ cells). CD45^-^ non-haematopoietic fractions of cells which encompass all known osteoprogenitor stem cells populations, were similar in control and treatment groups.

**Discussion:**

This study uncovers previously unidentified regulatory functions of BMP-6 and shows that BMP-6 enhances fracture healing by not only acting on osteoprogenitor stem cells but also by promoting type 2 immune response.

## 1 Introduction

Of the 7.9 million fractures occurring within the United States each year, roughly 400,000 to 1.6 million result in bony nonunions (1, 2). Current treatment options for high-risk fractures rely on autografts, allografts, synthetic bone graft substitutes, and FDA-approved bone morphogenetic protein-2 (BMP-2) based products. Severe shortages of autografts and allografts, donor site morbidity associated with autograft use, risk of disease transmission through allografts, lack of osteoinductive potential of synthetic grafts, and the requirement of very high doses of BMP-2 which can lead to undesirable side effects, necessitate further research on existing bone repair strategies (3–8).

BMP was discovered in 1965 by Marshall R. Urist and received FDA approval for clinical use in 2002. It was estimated in the following decade that the cumulative purchase cost of bone grafts, bone graft substitutes, and BMPs was 1.5 billion USD with BMPs accounting for 50% of that cost (3). Decades of research has provided useful insights on the BMP signaling pathway and how activation of the BMP pathway induces differentiation of stem cells (9, 10). However, BMP therapy still remains controversial (6–8). Adverse side effects arising from usage of high-dose BMP therapies have been reported – these include swelling, postoperative inflammation, ectopic bone formation, osteoclast-mediated bone resorption, and unwanted adipogenesis (11). As inflammation appears to be playing a major role in causing BMP-associated adverse effects, it is necessary to fully understand the effects of BMPs on the host immune system during BMP-induced bone formation, which has not yet been studied.

Some of the key strategies that are being investigated to eliminate these worrisome side-effects and to exploit full potential of BMPs are: use of low-dose BMP therapies, addition of vascular endothelial growth factor (VEGF), bisphosphonates, and anti-inflammatory drugs (11, 12). In this regard, our laboratory has used BMP-6 which is known to possess higher osteogenic potential than BMP-2. Our rationale for using BMP-6 is that BMP-mediated bone regeneration can likely be achieved with significantly lesser amount of BMP-6 than BMP-2 and this will likely avoid the high-dose associated side effects of BMP therapy. However, safety of low-dose BMP-6 therapy will gain real confirmation only after FDA’s approval for its therapeutic usage. We have also studied the cross talk between VEGF and BMP-6 signaling pathways in stem cells *in vitro* and *in vivo* and the effects of activation of hedgehog (Hh) signaling pathway on BMP-6 mediated bone regeneration (13–18). We found that a combination of BMP-6, VEGF, and smoothened agonist (SAG), an activator of the Hh signaling pathway, outperformed BMP-2 (11 µg, matching clinical doses, ~5-times higher dose than VEGF and BMP-6) in enhancing rat mandibular defect healing at week 8 (18).

We hypothesized that the cocktail or the individual ingredients will show anti-inflammatory activities, unlike the high dose BMP-2 therapy. Accordingly, in the present study the effects of BMP-6, VEGF, and SAG on the bone healing were evaluated at week 8 in correlation with cellular landscape of the fracture callus at week 2, with special reference to type 1 and type 2 immune responses. Difficulties associated with preparation of live single-cell-suspension, from the mineralizing calluses, have kept the investigators away from utilizing flow cytometry extensively to interrogate the cellular mechanisms of bone healing. The model system developed in this study consisting of a convenient bone defect model and a flow cytometry based protocol will likely be useful for other investigators for further research in this aspect. We have previously shown that the enhanced recruitment of T cells in the stem cells implants at week 1 as determined by flow cytometry, followed by enhanced expression of IFN-γ in the implants at weeks 3 and 6 as determined by real time PCR; inhibits the stem cells induced ectopic bone formation in mice at week 6 (19). Other investigators have used immunohistochemistry and shown that in mice maximum infiltration of T and B cells occurs at week 2 during femoral fracture healing (20). Based on these findings, the present study investigates immune response using flow cytometry at week 2 during rat mandibular defect healing.

This study was the first attempt of its kind to use a flow cytometry based approach to investigate immune response during rat mandibular defect healing. It was thus uncertain whether enough number of live and healthy cells could be obtained from mineralizing fracture callus to study a wide range of immune cells using a large set of surface and intracellular markers. Due to this uncertainty of cell yields, an antibody panel containing a selected number of essential markers was used to identify the maximum number of key immune cell types. Therefore, CD45, CD3, CD4, CD11b/c, CD38, IFN-γ, and IL-4 were used as markers in this study. Based on conventional identification and recent literature, this panel of antibodies could identify non-T cells compartment containing innate cells and B cells (CD45^+^CD3^-^), M1 macrophages (CD45^+^CD3^-^CD11b/c^+^CD38^hi^), M2 macrophages (CD45^+^CD3^-^CD11b/c^+^CD38^low^), T cells (CD45^+^CD3^+^), CD4^+^ T cells (CD45^+^CD3^+^CD4^+^), differentiated cells representing type 1 immune response - Th1 cells or M1 macrophages (CD45^+^CD4^+^IFN-γ^+^), differentiated cells representing type 2 immune response – Th2 cells, or M2 macrophages (CD45^+^CD4^+^IL-4^+^) (21–28). It could also suggest the presence of CD8^+^ T cells (CD45^+^CD3^+^CD4^-^), differentiated NK, NKT or cytotoxic T cells producing interferon gamma (CD45^+^CD4^-^IFN-γ^+^), activated mast cells, eosinophils or basophils producing IL-4 (CD45^+^CD4^-^IL-4^+^), T cells with regulatory functions (CD45^+^CD3^+^CD4^+^CD38^+^, CD45^+^CD4^-^IFN-γ^+^CD38^+^), M1 macrophages (CD45^+^CD3^-^CD11b/c^+^CD38^hi^, CD45^+^CD4^+^IFN-γ^+^) and M2 macrophages (CD45^+^CD3^-^CD11b/c^+^CD38^low^, CD45^+^CD4^+^IL-4^+^), using this set (21–28). Bone forming mesenchymal stem cells (MSCs) are defined as CD105^+^CD90^+^CD73^+^CD34^-^CD45^-^ (29). Therefore, the CD45- fraction could provide approximation for bone forming fraction of the cells.

## 2 Materials and methods

This study was conducted at the University of Virginia, Charlottesville, VA, from May 2019 to May 2021. Data obtained from fifty-four, 10-week-old, female, Lewis rats is reported in this study. Only female rats were used in this study as the experimental creation of atrophic nonunions is known to be reproducible only in female but not in male mice and rats (30, 31). Surgical procedures and all animal care was performed in accordance with the protocols and approvals of the University of Virginia Animal Care and Use Committee.

### 2.1 Scaffold preparation

The scaffolds were created using Matrigel^®^ Growth-Factor-Reduced basement membrane (Corning Inc., Corning, NY) and the growth factors VEGF: 2.5 µg (ProSpec, Rehovot, Israel), BMP-6: 2.5 µg (PeproTech, Rock Hill, NJ), and SAG: 0.25mg (Abcam, Cambridge, UK). As per the manufacturer’s technical datasheet matrigel contains bFGF 0-0.1 pg/mL, EGF < 0.5 ng/mL, IGF-1 5 ng/mL, PDGF < 5 pg/mL, NGF < 0.2 ng/mL, TGF-β 1.7 ng/mL. On the day of surgery, VEGF, BMP-6, and SAG were mixed with liquid matrigel aliquots on ice to yield 0.1 mL of scaffold which was then delivered to defect site and allowed to solidify. SAG dosing was 1.0 mM, significantly above the EC_50_ (half-maximal effective concentration) for SAG and was comparable to SAG concentration used by other investigators (32, 33).

### 2.2 Surgical procedure

Bilateral mandibular defects were created in all fifty-four rats as described previously (16, 18). In brief, non-continuity, critical-sized defects were made at the angle of the mandible with a 4-mm circular drill under general anesthesia (ketamine hydrochloride and xylazine, weight-based formula). Control scaffold and various combinations of treatment scaffolds containing BMP-6 (B), VEGF (V), and SAG (S), including control scaffold, B, V, S, BV, BS, VS and BVS, were delivered directly into the defect site using a pipette, allowed to solidify under surgical heat lamp, and then secured in the pterygomasseteric sling upon closure. Rats were administered buprenorphine (weight-based formula) for post-operative analgesia. Rats were given water with enrofloxacin (Bayer, Leverkusen, Germany) for one week post-operatively and kept on a soft food diet. No subjects were euthanized due to failure to thrive and there were no postoperative infections. One rat died from the BMP-6 treatment group at week 2 most likely due to inadequate oral intake secondary to post-surgical pain as the rat did not consume appropriate amount of food for consecutive 3 days when compared to other experimental animals in the study.

### 2.3 Radiography

Rats were euthanized at post-operative week 8 to measure bone formation. The mandibles were surgically removed and placed on Kodak X-ray film. Radiographs were taken with a Hewlett-Packard Faxitron (Series X-ray System, 43805N) with settings 32.5 kVp and 42 mAs. The films were subsequently developed and placed into a GS-800 Calibrated Densitometer (Bio-Rad, Hercules, CA) to convert the radiograph films into digital images. Blinded analysis of x-rays for four mandibles from each group was performed *via* x-ray image review. Area of mandibular defect bone regeneration was determined by the blinded reviewer and then quantified as a percentage of the original defect area using ImageJ-based software, FIJI (34).

### 2.4 Microcomputed tomographic (µCT) measurements

Four mandibles isolated from six selected treatment groups (untreated control, scaffold control, SAG, VS, BS, and BVS) underwent scanning and analysis using a high-resolution desktop µCT imaging system (µCT40, Scanco Medical AG, Brüttisellen, Switzerland). Scans were acquired using a 10 µm^3^ isotropic voxel size, 70 kVP, 114 μA, 200 ms integration time, and were subjected to Gaussian filtration and segmentation. Post-processing was performed to reorient the scans such that the z-axis of the scan was normal to the surface of the defect in the angle of the mandible. A circular region of interest of 4 mm in diameter was then centered over the defect and extended through the full thickness of the bone. A mineral density lower threshold of 500 mgHA/cm^3^ was used to segment bone from soft-tissue within the region of interest and the Scanco Evaluation program was used to measure bone volume (BV, mm^3^), tissue mineral density (TMD, mgHA/cm^3^), and porosity (percent) of the new bone that formed in the defect. It was assumed that any bone within the region of interest found through the above parameters was newly formed bone.

### 2.5 Flow cytometry

To obtain sufficient numbers of live cells, two calluses harvested from bilateral mandibular defects were combined together to prepare a cell suspension for flow cytometry. For each group, four rats (eight mandibles, n=4) were used in the study. Defect calluses were harvested and then bluntly cut into smaller pieces to increase the surface area. Resultant tissue was digested with 10 mL of 1.6 mg/mL Collagenase Type IV (ThemoFisher Scientific, Waltham, MA) in 1x Dulbecco’s phosphate-buffered saline (DPBS) solution (ThemoFisher Scientific, Waltham, MA) at 37 ^0^C for 2.5 hours. Afterwards, 10 µL of DNase I (Sigma Aldrich, St. Louis, MO) was added at room temperature. After 10 minutes, the mixture was passed through a 100-μm nylon strainer (ThemoFisher Scientific, Waltham, MA) and then centrifuged at room temperature until a cell pellet was obtained. This was re-suspended in 5 mL of complete medium [1 g/L Glucose Dulbecco’s Modified Eagle Medium (ThemoFisher Scientific, Waltham, MA) + 10% fetal bovine serum (ThemoFisher Scientific, Waltham, MA) + 1% 100x penicillin/streptomycin (ThemoFisher Scientific, Waltham, MA)] along with 1 μL of Cell Activation Cocktail with Brefeldin A (BioLegend, San Diego, CA). After incubation in the dark at 37°C for 1 hour, the mixture was centrifuged to obtain a cell pellet. A cell count was then obtained *via* cell counter (Scepter™ 2.0 Cell Counter, Millipore Sigma, Burlington, MA).

The cell pellet (~ 10^6^ cells on average) was resuspended in DPBS and equally redistributed into a 96-well plate on ice (~250,000 cells in each well on average). The cells were first blocked with 1 μL of 2.5 mg/mL Rat IgG (Invitrogen, Carlsbad, CA) for 20 minutes to prevent nonspecific binding of the antibodies to the cell surface. Centrifugation was repeated, followed by a single wash with DPBS and resuspended in 100 μL of DPBS. In the surface staining group, cells were incubated with 1 μL of each antibody against CD3 (0.2 mg/mL, PE conjugated, BioLegend, Clone: 1F4, San Diego, CA), CD4 (0.2 mg/mL, PE/Cyanine 7 conjugated, BioLegend, Clone: W3/25), CD45 (0.2 mg/mL, Brilliant Violet 421 conjugated, Clone: OX-1, BD, Franklin Lakes, NJ), CD38 (0.5 mg/mL, Fluorescein isothiocyanate conjugated, BioLegend, Clone: 14.27), CD11b/c (0.5 mg/mL, Alexa Fluor 647 conjugated, BioLegend, Clone: OX-42) or with their corresponding isotype controls (PE conjugated Mouse IgG1 κ, 40 μg/mL, Clone: MOPC-21; PE/Cyanine 7 conjugated Mouse IgG1 κ, 200 μg/mL, Clone: MOPC-21; Brilliant Violet 421 conjugated Mouse IgG1 κ, 100 μg/mL, Clone: MOPC-21; FITC conjugated Mouse IgG2b κ, 0.5 mg/mL, Clone: MPC-11; Alexa Fluor 647 conjugated Mouse IgG1 κ, 50 μg/mL, Clone: MOPC-21; BioLegend, San Diego, CA) in the control group for 30 minutes. Anti-rat CD45 antibody was used in the control group rather than its isotype control antibody to facilitate background staining on CD45 hierarchal analysis.

In the intracellular group, cells were incubated with 1 μL of each antibody against CD4, CD38, CD45 or with their corresponding isotype controls in the control group for 30 minutes. Anti-rat CD4 and CD45 antibodies were used in the control group rather than their isotype control antibodies to facilitate background staining on CD45 and CD4 hierarchal gating.

In both groups, after washing with DPBS solution, cells were resuspended in 100 μL of DPBS and stained with 1 μL of Live/Dead™ Fixable Near-IR dye in 50 μL DMSO (ThemoFisher Scientific, Waltham, MA) for 20 minutes following the manufacturer’s protocol. After washing twice, cells in each well were incubated with 100 μL of Fixation/Permeabilization solution (BD, Franklin Lakes, NJ) for 20 minutes. Cells were then washed with 100 μL of 1x Permeabilization Wash solution (BD, Franklin Lakes, NJ), twice and resuspended in 100 μL of 1x Permeabilization Wash solution. After this step, the surface marker groups were ready for analysis. The intracellular staining groups were further incubated with 1 μL of each antibody against IFN-γ (50 μg/mL, Alexa Fluor 647 conjugated, BioLegend, Clone: DB-1) and IL-4 (0.2 mg/mL, PE conjugated, BioLegend, Clone: OX-81) for 30 minutes in the 1x Permeabilization Wash solution. The control groups were stained with the corresponding isotype controls (25 μg/mL, Alexa Fluor 647 conjugated Mouse IgG2a κ, Clone: MOPC-173, and 0.2 mg/mL, PE conjugated Mouse IgM κ, Clone: MM-30, both BioLegend). After the incubation, cells were washed with 1x Permeabilization Wash solution once and stored in the 1x Permeabilization Wash solution at 4 °C in the dark until flow cytometry analysis.

Before analyses, the 96-well plate was centrifuged and the resulting cell pellet in each well was resuspended in 350 μL of FACS buffer [1x DPBS + 1% fetal bovine serum + 0.01% sodium azide (Millipore Sigma, Burlington, MA)]. Samples were acquired in the Flow Cytometry Core Facility on an Attune NxT Flow Cytometer (Thermofisher) equipped with 4 laser lines (405nm 100mw, 488nm 100mw, 561nm 100mw, 637nm 140mw). The data was analyzed with FCS Express 6 software (*De Novo* Software, Pasadena, CA).

### 2.6 Statistical analysis

GraphPad Prism 8 (GraphPad Software, La Jolla, CA) was used for statistical comparison analysis of the acquired x-ray, μCT, and flow cytometry data using one-way analysis of variance (ANOVA) followed by Tukey’s multiple comparisons test.

## 3 Results

### 3.1 BMP-6 treatment heals critical sized rat mandibular defect at week 8

**Figure 1:**
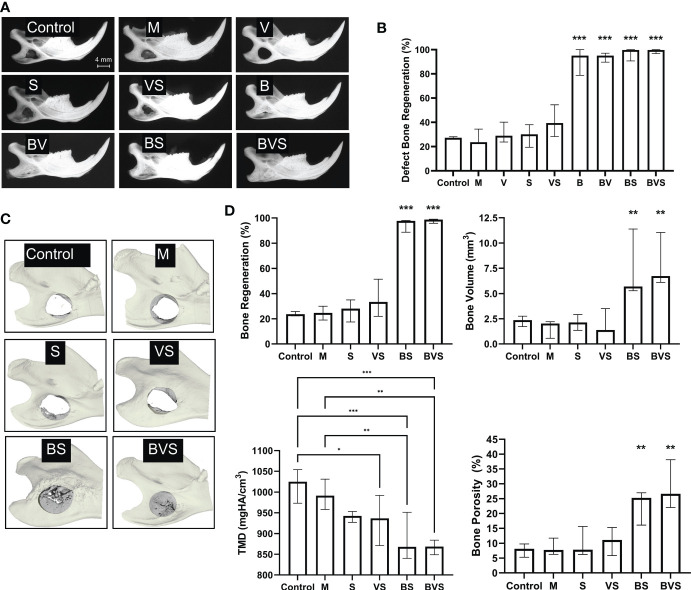
BMP-6 treatment significantly enhances bone regeneration in critical-sized rat mandibular defect to attain complete bony union. Radiography **(A)** and µCT **(C)** measurements at post-operative week 8. **(A)** Representative radiographic images of harvested mandibles at week 8, **(B)** Percentage of defect bone regeneration from blinded analysis of plain film radiographs of explanted mandibles from all groups, **(C)** Representative 3D-reconstructions of µCT measurements of selected groups (untreated control, Matrigel control, S, VS, BS, and BVS), and **(D)** Percent bone regeneration, bone volume, tissue mineral density (TMD), and porosity of newly formed bone within the 4 mm diameter area of original defect in groups shown in panel **(C)** as determined by µCT measurements. Statistical significance: * (P ≤ 0.05), ** (P ≤ 0.01), *** (P ≤ 0.001).

BMP-6 lacking groups – untreated control, matrigel control, S, V, and VS – did not show appreciable healing with mean bone generation rates being between 20% to 40% (**Figures 1A, B**). In contrast, radiographs clearly showed that every treatment group containing BMP-6 – B, BV, BS and BVS induced significant bone regeneration to demonstrate bone healing of greater than 90% at week 8 (**Figures 1A, B**). Across all the treatment groups, the BVS group showed the maximum bone healing of 99.06% (**Figure 1B**). High resolution µCT confirmed that the groups lacking BMP-6 demonstrated poor healing outcomes (20-30% on average) and remained as nonunions at week 8 (**Figures 1C, D**). µCT analysis also confirmed that the bone regeneration percentages obtained through radiography were accurate.

### 3.2 Preparation of single-cell-suspension from fracture callus and gating strategy

**Figure 2:**
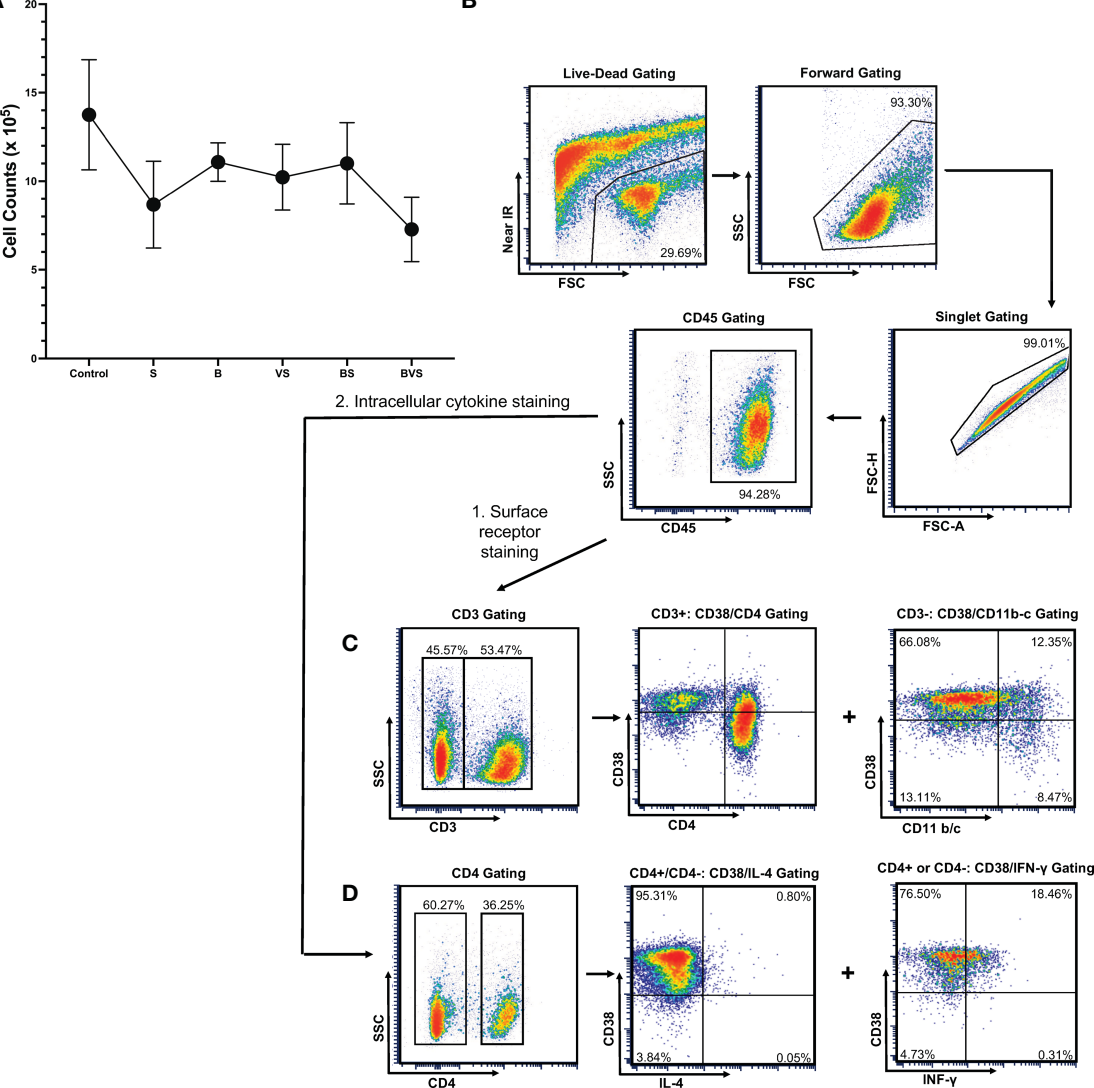
Gating strategy for the live cells harvested from bony calluses to perform flow cytometry measurements. The cells were harvested from bony calluses of control and treatment groups at post-operative week 2. **(A)** Cell yield from control and treatment groups. **(B)** Live cells were gated first with live-dead infrared discrimination stain, followed by lymphocyte gating based on cell size and granularity, and then singlet gating. CD45^+^ cell populations were then selected for further analyses. **(C)** Gating strategy for surface receptor staining. **(D)** Gating strategy used for intracellular cytokine staining.

After digestion of bony calluses for 2.5 hours, ~ 1 million cells were obtained from all the groups - the control groups as well as the treatment groups. Maximum cell yield of 1.4 x 10^6^ was obtained from the matrigel control group, while the BVS treatment group gave the minimum cell yield of 0.7 x 10^6^ cells (**Figure 2A**). Thus, there was not much variation in the cell yields.

The gating strategy is shown in the figure using the VS group as a representative example (**Figure 2B**). Staining with Live/Dead™ Fixable Near-IR Dead Cell Stain Kit and flow cytometry revealed that 60% of the cells obtained were alive and healthy. Using a combination of live, lymphocyte and singlet gates, a population of CD45^+^ cells was defined after staining the cells with anti-CD45 antibody and was used to determine percentages of downstream sub-types of immune cells at week 2 (**Figures 2C, D**).

### 3.3 BMP-6 treatment significantly increases CD4/CD8 T-cell ratio in the fracture callus at week 2 during bone regeneration

**Figure 3:**
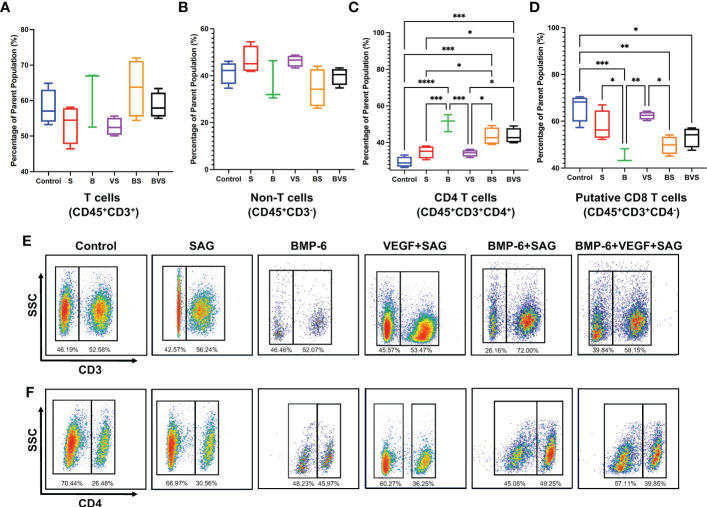
BMP-6 modulates ratios of CD4/CD8 T-cells in the fracture calluses at day 14 during rat mandibular defect healing. Cells were harvested from the fracture calluses, stained with specific antibodies, and then analyzed using a flow cytometer to measure proportions of **(A)** T-cells, **(B)** non-T cells, **(C)** CD4+ T-cells, **(D)** Putative CD8+ T-cells. **(E)**, **(F)** Dot plots showing staining of the cells with antibodies for CD3 and CD4 in control and treatment groups. Statistical significance: * (P ≤ 0.05), ** (P ≤ 0.01), *** (P ≤ 0.001).

The proportions of T-cells (CD45^+^CD3^+^) and non-T cells (CD45^+^CD3^-^) were comparable in the fracture calluses of all the treatment groups and control at week 2 (**Figures 3A, B, E**). ~60% of the CD45^+^ cells were found to be T cells at this time point while remaining ~ 40% were non-T cells (innate cells and B cells). BMP-6 treatment significantly increased proportions of CD4^+^ T cells (CD45^+^CD3^+^CD4^+^) while significantly reducing the proportions of putative CD8^+^ T cells (CD45^+^CD3^+^CD4^-^) (**Figures 3C, D, F**). Interestingly, this effect was observed in all the BMP-6 containing treatment groups – B, BS, and BVS, indicating that BMP-6 could override effects of S and V showing strongest influence on CD4T/CD8T cells ratio in the fracture callus at week 2.

### 3.4 BMP-6 treatment does not regulate type 1 immune response in the fracture callus at week 2 during bone regeneration

**Figure 4:**
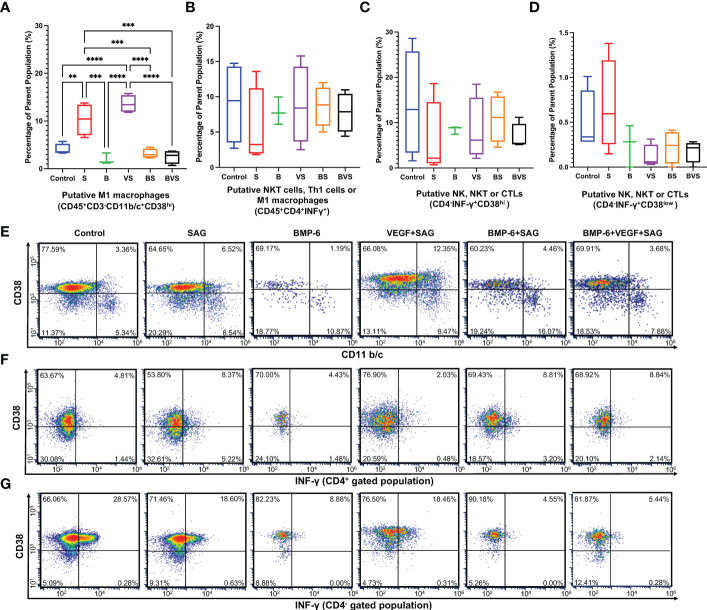
BMP-6 does not alter type 1 immune response in the fracture callus at day 14 during rat mandibular defect healing. Cells were harvested from the fracture calluses, stained for surface receptors, permeabilized, stained for intracellular cytokines, and then analyzed using a flow cytometer to measure proportions of **(A)** M1 macrophages, **(B)** Putative - Th1 cells or M1 macrophages, **(C,D)** NK, NKT or cytotoxic T cells (CTLs). **(E–G)** Dot plots showing staining of the cells for surface expression of CD11b/c and intracellular IFN-γ. Statistical significance: ** (P ≤ 0.01); *** (P ≤ 0.001); **** (P ≤ 0.0001).

IFN-γ which is signature cytokine of type 1 immune response, is produced by only NK, NKT, M1 macrophages, Th1 cells and cytotoxic T cells (CTLs). Both macrophages and CD4+ T cells in rat express CD4. However, NK cells as well as cytotoxic T cells do not express CD4 while NKT cells may or may not express CD4. CD38 is recently identified as M1 macrophage specific marker in mice and humans. Based on these facts following putative subsets producing IFN-γ were identified in **Figure 4**–M1 macrophages, Th1 cells, NK, NKT and CTLs. Numbers of CD45^+^CD3^-^CD11b/c^+^CD38^hi^ M1 macrophages were significantly increased in S and VS groups in comparison with the control (**Figures 4A, E**). Increases proportions of M1 macrophages in S and VS groups corresponded to poor healing outcomes at week 8 (**Figure 1**). BMP-6 containing groups showed significantly less proportions of M1 macrophages in comparison with S and VS groups, but there was no statistically significant difference between BMP-6 containing groups and the control. Interrogation of IFN-γ production from CD45^+^CD4^+^, CD45^+^CD4^-^CD38^hi^, and CD45^+^CD4^-^CD38^low^ populations revealed that there were no differences across the groups (**Figures 4B–D, F, G**)

### 3.5 BMP-6 promotes type 2 immune response at week 2 during bone regeneration

**Figure 5:**
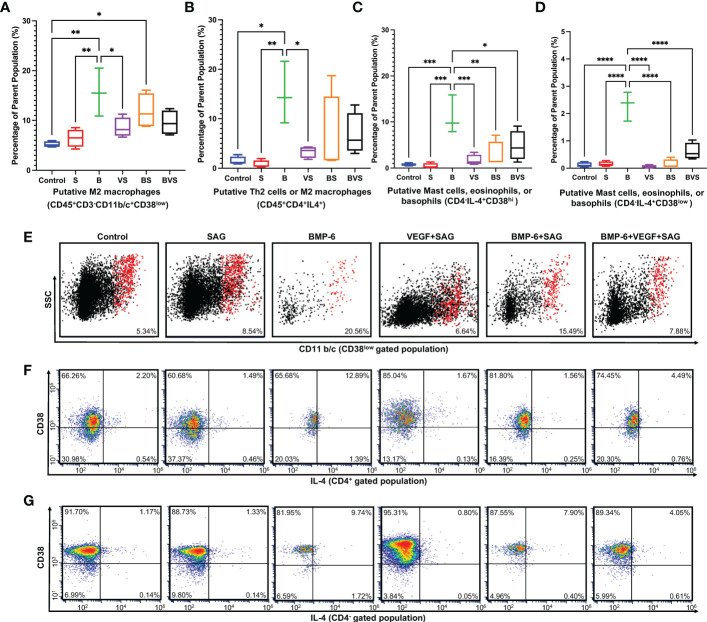
BMP-6 promotes type 2 immune response in the fracture callus at day 14 during rat mandibular defect healing. Cells were harvested from the fracture calluses, stained for surface receptors, permeabilized, stained for intracellular cytokines, and then analyzed using a flow cytometer to measure proportions of **(A)** M2 macrophages, **(B)** Putative – Th2 cells or M2 macrophages, **(C, D)** Putative- Mast cells, eosinophils or basophils. **(E–G)** Dot plots showing staining of the cells for surface expression of CD11b/c and intracellular IL-4. Statistical significance: * (P ≤ 0.05); ** (P ≤ 0.01); *** (P ≤ 0.001); **** (P ≤ 0.0001).

IL-4 is the signature cytokine of type 2 immune response and is produced by only mast cells, eosinophils, basophils, Th2 cells and M2 macrophages. Both macrophages and CD4+ T cells in rat express CD4. Mast cells, eosinophils and basophils do not express CD4. CD38 is recently identified as M1 macrophage specific marker in mice and humans. Based on these facts following putative subsets producing IL-4 were identified in **Figure 5**–M2 macrophages, Th2 cells, mast cells, eosinophils and basophils.

Proportions of both the CD45^+^CD3^-^CD11b/c^+^CD38^low^ M2 macrophages and CD45^+^CD4^+^IL-4^+^ Th2 cells were significantly enhanced upon BMP-6 treatment at week 2 (**Figures 5A, B, E, F**). Interestingly, this effect was seen only in the BMP-6 alone group indicating that VEGF and SAG from BS and BVS groups retarded the BMP-6 enhancement of type 2 immune response. This is also corroborated by the finding that S and VS groups showed significantly higher numbers of M1 macrophages (**Figure 4**). IL-4 is produced by Th2 cells, mast cells, eosinophils and basophils. BMP-6 significantly increased IL-4 production in CD45^+^CD4^+^, CD45^+^CD4^-^CD38^hi^ and CD45^+^CD4^-^CD38^low^ populations at week 2 (**Figures 5C, D, F, G**).

### 3.6 Recruitment of CD45^-^ cells during BMP-6 mediated bone healing

**Figure 6:**
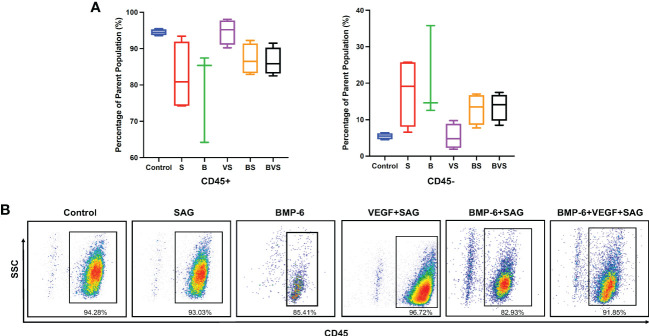
Recruitment of CD45- cells containing all known skeletal stem cells populations at day 14 during rat mandibular defect healing. Cells were stained with anti-CD45 antibody and then analyzed using flow cytometer. **(A)** Proportions of CD45- fractions of cell population, **(B)** Dot plot of cells stained for expression of CD45 receptor.

As there were no stem cells added exogenously, all the bone formation that occurred in the treatment groups depended on the endogenous osteoprogenitors stem cells. The non-haematopoietic CD45^-^ cell population is known to contain mesenchymal stem cells. At week 2, ~ 90% of the total live cells were CD45^+^ whereas only ~ 10% of the cells were CD45^-^ in all the groups (**Figure 6**). There was no statistically significant difference in numbers of CD45^-^ cells recruited to the bone defect at week 2, between any of the treatment groups and the control.

## 4 Discussion

The term “osteoimmunology” was coined in the year 2000 to systematically initiate the studies on the effects of the immune system on bone development, homeostasis and repair (35). Although rapid progress has been made in this relatively new field, the roles of various immune cell types during the bone healing process are still incompletely understood. Most of the work pertaining to this subject has relied on studying fracture healing rates in mouse models lacking macrophages or T-cells (36–39), investigation of the bone healing after adoptive transfer of T-cells (40, 41), and immunostaining of the fracture callus (20, 42). While these methodologies provided meaningful insights on the role of the immune system in bone healing, they were indirect, with low resolution and semi-quantitative in nature as they did not utilize the reliable approach of flow cytometry to identify and to quantitate numerous sub-types of immune cells directly from the fracture callus during the bone healing process. The current study presents a flow cytometry based model system to address this limitation and subsequently, the model can be adapted for future investigations to precisely identify various subsets of the immune cells and their functions during the bone healing process.

Fracture healing has been shown to be accelerated in Rag 1-/- mice, which lack T-cells and B-cells. mRNA expression of IFN-γ, an inflammatory cytokine which is the hallmark of type 1 immune response, was significantly higher at the fracture repair site in wild type mice; whereas the expression of anti-inflammatory cytokine IL-10, was significantly enhanced in Rag 1-/- mice (37). Mice deficient in γδ T-cells have been shown to produce significantly decreased amounts of IFN-γ and IL-6 at the fracture repair site in comparison with wild type mice, which creates the bones with improved stability and superior biomechanical strength (36). These studies demonstrated that the T-cells mediated type 1 immune response inhibited bone healing. In agreement with this notion, adoptive transfer of T regulatory (Treg) cells, which are known to inhibit inflammatory T-cell activities including the type 1 immune response, significantly improved fracture healing (40). In a fracture model in rodents, maximum infiltration of fracture callus by T and B cells was reported to occur on day 14 (20).

In opposition to the notion that T-cells in general inhibit fracture healing (36, 37) and in contradiction with earlier findings (37), it was demonstrated that Rag 1-/- mice display delayed fracture healing owing to lack of T-cell derived IL-17 and it was subsequently proposed that Th17 cells enhance fracture healing (38). Newly formed bone in Rag 1 -/- mice was found to be stiffer and thus did not have the elasticity to absorb forces, which is crucial for fracture prevention. This was a consequence of dysregulated collagen deposition and osteoblast distribution in absence of T-cells (39). These studies suggest that role of various sub-types of T cells during fracture healing needs further investigation. Our study clearly demonstrates that type 1 immune response at day 14 does not inhibit the fracture healing.

The present study also shows that a high CD4^+^/CD8^+^ T-cell ratio, higher percentages of CD45^+^CD3^-^CD11b/c^+^CD38^low^ M2 macrophages, CD45^+^CD4^+^IL-4^+^ cells (putative - Th2 cells or M2 macrophages), and CD45^+^CD4^-^IL-4^+^ cells (putative – mast cells, eosinophils, basophils) at day 14 are the hallmarks of successful bone healing and can be used as prognostic indicators for successful outcome of the therapy. It is likely that a higher CD4+/CD8+ T-cell ratio indicates the higher proportion of CD4+CD25+FoxP3+ Treg cells at the fracture callus that are known to promote bone formation. Higher percentages of three subsets of CD8^+^ T-cells – CD11a^+^, CD57^+^, and CD28^-^ – within patients’ peripheral blood has been reported to correlate with delayed fracture healing (43). It will be essential to determine whether the immune cells subsets identified in this study also show similar patterns in the peripheral blood, to use them as prognostic indicators of successful fracture healing.

Macrophages are known to play an important role during bone development, homeostasis, and repair (44–48). Macrophage depletion results in retardation of skeletal growth, progressive osteoporosis, 60% reduction in the number of bone marrow mesenchymal progenitor cells, and also leads to impairment of fracture healing (44). M1 and M2 macrophages are required during pro-inflammatory and ossification phases of the fracture repair process, respectively (46, 48). The present study demonstrates that the presence of M2 macrophages at day 14 is essential prerequisite for successful fracture healing outcomes.

The present study provides the first direct evidence that BMP-6 either directly modulates the cellular milieu of the bone healing environment to promote the type 2 immune response or triggers certain cells to indirectly promote the type 2 immunity. BMPs are known to play a role in patterning and cellular fate determination in various organs, including the thymus; furthermore, thymocytes, as well as matured T-cells, are known to express BMP receptors (49–52). However, the precise function of BMPs and their receptors in governing immune cells functions is not fully understood. A recent discovery revealed that BMPs are immunoregulatory cytokines and can induce formation of Foxp3+ Treg cells (53). In agreement with this recent astonishing discovery, the data presented here shows that BMP-6 plays an immunoregulatory role during fracture healing.

BMP receptors are expressed in macrophages and activation of BMP pathways has been reported to modulate macrophage differentiation (54–58). It has been reported that BMP stimulation activates macrophages to produce inflammatory cytokines IL-1, IL-6, TNF-α, and iNOS (54, 55, 57). Contradictorily, it has also been shown that the BMP co-receptor DRAGON is a negative regulator of IL-6 production in macrophages and that BMPs inhibit M1 macrophages (56, 58). The data presented in the present study clearly revealed that BMP-6 enhances M2 macrophages to promote bone healing.

The pleiotropic role of BMP-6 identified in this study shows that BMP-6 can not only enhance differentiation of osteoprogenitors, but can also modulate T-cells and macrophages. Recent advances in osteoimmunology suggest an important role of immune cells in the bone healing process, various bone diseases such as osteonecrosis, and in regulating the side effects of currently used high-dose BMP therapy for fracture repair; therefore, the pleotropic role of BMPs needs urgent further investigation. It is also necessary to identify all possible sub-types of immune cells that are potential targets of BMP, their temporal activation status during fracture healing, and the role of these sub-types in the craniofacial skeleton versus long bones.

A more active immune microenvironment is identified in the craniofacial skeleton where a significantly higher proportion of mature immune cells are present than in long bones (59). Macrophages from the craniofacial skeleton actively interact with stem cells to promote their osteogenic differentiation. Similarly, γδ T-cells are likely to play a protective role in the craniofacial skeleton by preventing bisphosphonate associated osteonecrosis (60, 61). However, T-cells did not seem to play any role, protective or otherwise, in ovariectomy induced bone loss in the long bones of mice (62). The presented study underscores the importance of the immune system in skeletal health and the potential of BMP-6 for the repair of osseous defects in the skeletal system through its pleotropic functions reported in this paper.

Although bone morphogenetic proteins have been used as key therapeutic strategy in clinical practice for treatment of difficult bone fractures, their physiological effects on the host immune system and subsequently, bone healing, have never been elucidated. In this study, it was discovered that low dose BMP-6 therapy did not induce any undesirable inflammatory effects, that are reported with high dose BMP-2 therapy currently being used in the clinic for the treatment of bone defects and complete healing of the bone defect was achieved at week 8. Remarkable cell recruitment patterns were observed under the influence of BMP-6 at week 2 that could serve as prognostic biomarkers for predicting long-term bone healing outcomes. For the first time, the present study uncovers immunomodulatory functions of BMP-6 during bone regeneration and demonstrates that BMPs have a pleiotropic role during the bone healing process.

## Data availability statement

The original contributions presented in the study are included in the article/supplementary material. Further inquiries can be directed to the corresponding author.

## Ethics statement

The animal study was reviewed and approved by University of Virginia Animal Care and Use Committee.

## Author contributions

Conceptualization: LM, XC, QC, AD. Investigation: LM, XC, MS, KS, JC, QC, AD. Formal analysis: LM, XC, MS, RC, AD. Methodology: LM, XC, MS, SH, KF, EZ, AD. Resources: PL, EZ, SP, JC, QC, AD. Funding acquisition: JC, QC, AD. Data curation and visualization: LM, XC, MS. Writing: LM, XC, PL, EZ, QC, AD. All authors contributed to the article and approved the submitted version.
